# Pseudoaneurysmal rupture with massive bleeding following endoscopic ultrasound-guided gallbladder drainage using an electrocautery-enhanced lumen-apposing metal stent

**DOI:** 10.1055/a-2578-2805

**Published:** 2025-04-28

**Authors:** Kyong Joo Lee, Se Woo Park, Dong Hee Koh

**Affiliations:** 1 366256 Gastroenterology, Department of Internal Medicine, Hallym University Dongtan Sacred Heart Hospital, Hwaseong-si, Korea (the Republic of)

Endoscopic ultrasound-guided gallbladder drainage (EUS-GBD) is a minimally invasive and effective treatment for patients at high risk of acute cholecystitis who are unsuitable for surgical intervention due to underlying comorbidities 1 . Although EUS-GBD is generally considered safe, with reported bleeding events in 2.1–4.3% of cases 2 , the occurrence of pseudoaneurysms is rare 3 . We report a case of pseudoaneurysmal rupture following EUS-GBD using a novel electrocautery-enhanced lumen-apposing metal stent (LAMS).

A 74-year-old woman with multiple myeloma, chronic kidney disease, and recurrent dyspnea presented with abdominal pain and developed acute cholecystitis 3 days after undergoing endoscopic retrograde cholangiopancreatography (ERCP) for bile duct stones. She exhibited a positive Murphyʼs sign. Abdominal computed tomography (CT) revealed gallbladder dilation with pericholecystic fluid collection. Given her poor surgical candidacy, EUS-GBD was performed ( Video 1 ).

Inadvertent injury to the opposite gallbladder wall caused by puncture during endoscopic ultrasound-guided gallbladder drainage (EUS-GBD) in a 74-year-old woman. Bleeding occurred after the deployment of the electrocautery-enhanced lumen-apposing metal stent but resolved spontaneously.Video 1

Using a linear echoendoscope (EG-580UT; Fujifilm Medical Systems, Tokyo, Japan), the gallbladder neck was punctured with a 19-gauge needle (EZ Shot3; Olympus Medical, Japan); however, the opposite gallbladder wall was inadvertently punctured. Following guidewire placement and contrast administration, the electrocautery-enhanced-LAMS (Niti-S HOT SPAXUS; Taewoong Medical, Goyang, Korea) was successfully deployed, resulting in substantial bleeding into the duodenum. The hemorrhage resolved spontaneously, and the patient was discharged on post-procedural day 3.

After 6 days, the patient was readmitted with jaundice and fever. Urgent ERCP revealed blood clots draining from both the bile duct and through the LAMS. Continuous drainage of fresh blood from the LAMS ( Fig. 1 **a** ) prompted an immediate CT scan, which showed high-density material within the gallbladder consistent with blood, along with a pseudoaneurysm on the gallbladder wall ( Fig. 1 **b** ). Angiography confirmed involvement of the cystic artery ( Fig. 2 **a** ), and embolization was successfully performed using gelfoam particles ( Fig. 2 **b** ). The patient’s symptoms resolved without further complications.

**Fig. 1:**
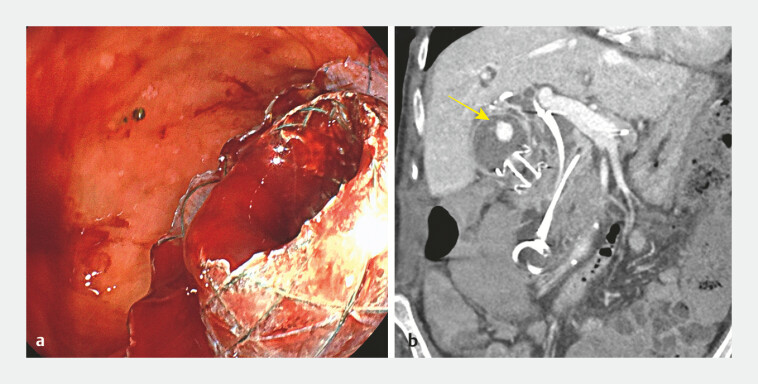
Endoscopic and radiologic findings of pseudoaneurysm following endoscopic ultrasound-guided gallbladder drainage. **a** Endoscopic view showing continuous fresh blood draining through the lumen-apposing metal stent into the duodenum. **b** Computed tomography scan revealing high-density material in the gallbladder (yellow arrow), indicating the presence of a pseudoaneurysm.

**Fig. 2:**
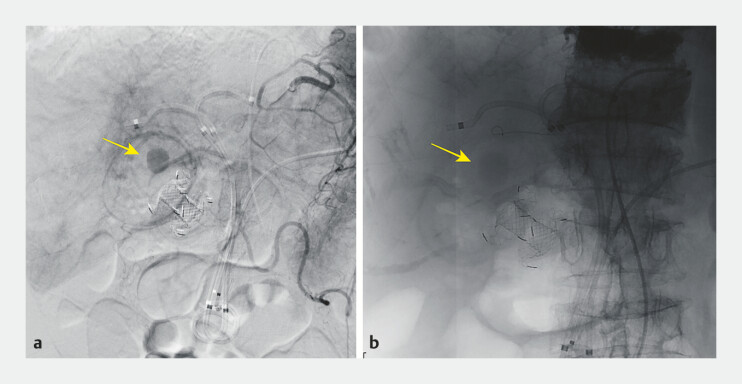
Angiographic findings and embolization of the cystic artery. **a** Angiographic image showing the presence of a pseudoaneurysm involving the cystic artery (arrow). **b** Angiographic image after gelfoam particle embolization showing successful resolution of the pseudoaneurysm (arrow).

The pseudoaneurysm was likely caused by mechanical trauma during the initial needle puncture. To reduce the risk of such vascular injuries, careful puncture of the gallbladder wall adjacent to the duodenum is essential.

Endoscopy_UCTN_Code_CPL_1AL_2AD

## Notes

Endoscopy E-Videos https://eref.thieme.de/e-videos
*E-Videos* is an open access online section of the journal *Endoscopy* , reporting on interesting cases and new techniques in gastroenterological endoscopy. All papers include a high-quality video and are published with a Creative Commons CC-BY license. Endoscopy E-Videos qualify for HINARI discounts and waivers and eligibility is automatically checked during the submission process. We grant 100% waivers to articles whose corresponding authors are based in Group A countries and 50% waivers to those who are based in Group B countries as classified by Research4Life (see: https://www.research4life.org/access/eligibility/ ). This section has its own submission website at https://mc.manuscriptcentral.com/e-videos .
